# Periodic almost-Schrödinger equation for quasicrystals

**DOI:** 10.1038/srep11492

**Published:** 2015-07-24

**Authors:** Igor V. Blinov

**Affiliations:** 1 Moscow Institute of Physics and Technology, 9 Institutskiy per., Dolgoprudny, Moscow Region, 141700, Russian Federation.

## Abstract

A new method for finding electronic structure and wavefunctions of electrons in quasiperiodic potential is introduced. To obtain results it uses slightly modified Schrödinger equation in spaces of dimensionality higher than physical space. It enables to get exact results for quasicrystals without expensive non-exact calculations.

Quasicrystals after Dan Shechtman’s^1^ discovery became that missing link between periodic crystals and amorphous solids. The new state of matter was in focus of particular interest because of the interesting electronic^2^, magnetic^3^, and elastic properties. Just as periodic crystals, quasicrystals have discrete diffraction pattern^1^, though it combined with the lack of periodicity^1^,^4^, which allows quasicrystals to have forbidden for periodic crystals symmetries.

An obvious idea for describing quasiperiodic crystals is to use so called crystalline approximants - periodic structure with a very large unit cell which emulates a quasiperiodic structure.

Another idea was developed in work of KKL^5^ where they used normalization method for a one-dimensional crystal and obtained first exact result for quasiperiodic structures. Unfortunately, there are no exact results for higher dimensional quasicrystals.

Another obvious idea for solving the electronic structure of quasicrystals is to use quasiperiodic functions. Or, more precisely, their periodic images embedded in higher-dimensional spaces.

This idea follows from the fact that qusiperiodic functions satisfy the desired properties: they are non-periodic and their diffraction pattern is discrete^6^,^7^.

This approach has a number of advantages:

1. both numerical and analytical calculations are almost as easy as for periodic structures.
2. errors of numerical calculations are related only to machine precision.

There were several successful attempts to use this method for different purposes (for example^8^,^9^,^10^, and more recent^11^). While other methods using slicing procedure applicable only to one-dimensional quasicrystals or to specific potentials, this article suggests completely general method for any physical space (1,2 or 3-dimensional) and any smooth potential. Proof of the method is also presented.

In this work I have tried to suggest completely multidimensional description for electrons in quasiperiodic potentional. This work hopefully will put a foundation for stable quasicrystals structure prediction.

## Quasiperiodic functions

Firstly, let me introduce a mathematical apparatus used in this work. Continuous quasiperiodic functions might be presented in Fourier series:


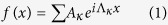


where 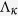, called Fourier indexes are real. Unlike periodic functions, where these numbers are integers, in almost periodic functions they might be even dense. And


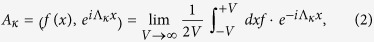


(see Ref. 6 or 7).

**Figure 1:**
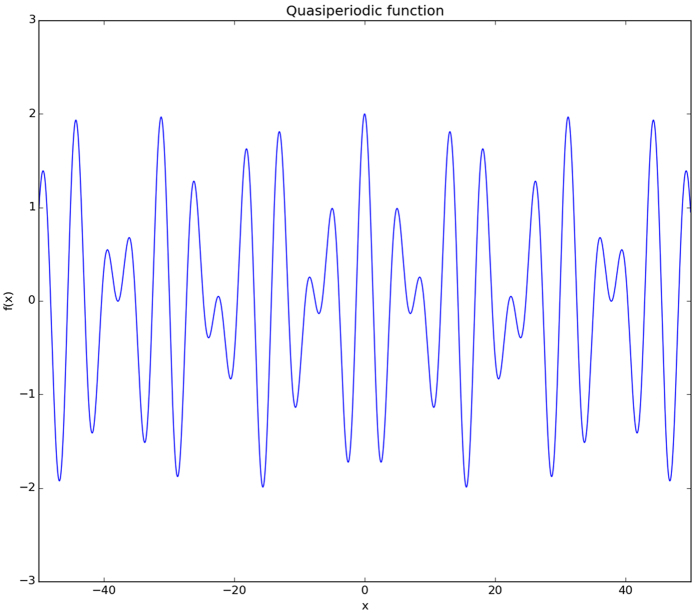
Quasiperiodic function 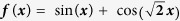.

**Figure 2:**
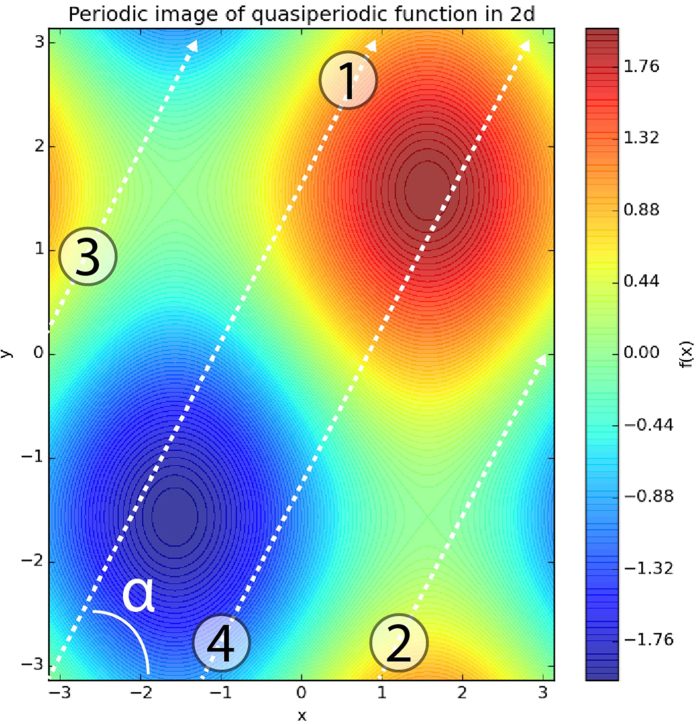
Periodic image of 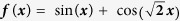. Moving along these and latter arrows (1 → 2 → 3 → 4 →…) quasiperiodic function might be generated. Tangent of *α* is equal to 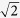 in particular or *τ* in general case.

It is widely known, that these functions are images of periodic functions on the space of higher dimension. For example, we may take simple quasiperiodic 1-dimensional function with two quasiperiods (Fig. 1)


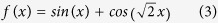


and, with transformation 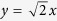 perform it as periodic in 2-dimensional space (Fig. 2).

## Physical Intuition

As it was said, we can embed a quasiperiodic one-dimensional function with two incommensurate periods in a two-dimensional space with transformation *y* = *τx*, where *τ* is a ratio of quasiperiods. Geometrical sense of *τ* is a tangent of the angle between x-axis and slicing line (Fig. 2). So, our transformation (x × y) → x looks like moving along the line *y* = *τx* and “measuring” value of our function. If we will try to imagine, how it looks in a unit cell, this should be moving along family of lines *y* = *τx + η*. The exact position on fixed line will be given by 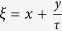.

The same “measuring” of a value of potential is what actually electron do. Embedded in a unit cell [−*a*, *a*] × [−*b*, *b*], its movement may look like going from (0, 0) along the line *y* = *τx*, then, on the top border *y* = *b* (if 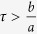), it will jump to the bottom border without changing x. After that, it will start going by another line and so on (see Fig. 2).

So, it might be reasonable to look for an equation connecting a two-dimensional image of one-dimensional wave function and periodic image of our quasiperiodic potential, in a form of


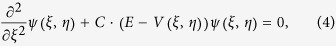


where *E* is the energy, *C* is some unknown constant, *ξ* and *η* are equal to 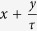 and 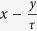 respectively.

## Mathematical proof

Quasiperiodic and periodic functions both might be expanded in Fourier series. Using this fact, we will prove an equivalence between (4) and one-dimensional Schrödinger equation with quasiperiodic potential.

### In one-dimensional space

Acting like in Bloch theorem, substitute 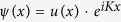, where *u*(*x*) is quasiperiodic with quasiperiods the same as potential has, then from ordinary Schrödinger equation we may obtain an equation for *u*(*x*):


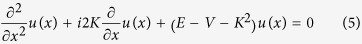


Now expand *V*(*x*)


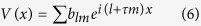


and *u*(*x*) as quasiperiodic functions. Using previously defined scalar product (2), we finally get:


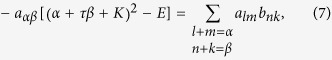


where coefficients *a*_*lm*_ and *b*_*nk*_ refer to *u*(*x*) and *V*(*x*) respectively.

### In two dimensions

As previously, we firstly make a substitution


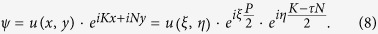


where *P* = *K* + *τN* and *u*(*x*, *y*) is periodic. Substituting this to (4), we obtain


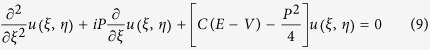


Then, making orthogonalization procedure with standard scalar product in *L*^2^,


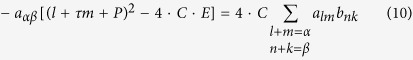


Comparing with (7) we may conclude that *C* must be equal to 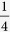 and, finally, our equation takes the form


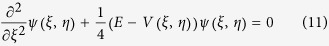


and is totally equivalent to the Schrödinger equation in one-dimensional space and quasiperiodic potential.

## Generalization

Using Fourier series, it is easy to generalize our equation to quasicrystals with higher dimensionality and arbitrary symmetries.

Firstly, let us expand our three dimensional function into series


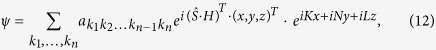


where, indexes *k*_*i*_ are integers corresponding to n-dimensional reciprocal vector H and *Ŝ* is the transformation matrix performing the slicing operation^12^


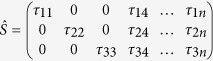


Each line in *Ŝ* represents basis vector for slicing space. In other words, matrix *Ŝ* it is a set of vectors defining slicing subspace. So, the most general form of kinetic part is exactly:


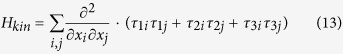


where *x*_*i*_ are the coordinates in high-dimensional space. This equation, suitable for any quasiperiodic potentials is the main result of this work (Sketch of the proof one can find in Appendix).

It is easy to show that latter equation is equivalent to previously introduced one-dimensional equation with two quasiperiods. In that particular case slicing space is a line (Fig. 2) given by vector


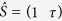


Kinetic part of the hamiltonian will look like


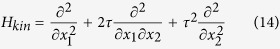


By substitution 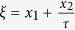 and 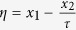 we will come to (11).

Equation can be also applied to objects periodical in one or several dimensions. We just need to zero out corresponding elements in *Ŝ*. As an example, we may do it for an object that has 12-fold symmetry in x-y and periodic along z-axis. In this specific case^12^, we will have *Ŝ* equal to


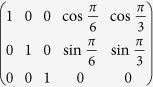


Starting from here one may find the kinetic part of our Hamiltonian


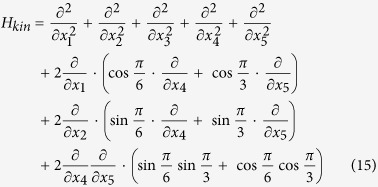


## Real systems

Imagine we have a solid with two types of atoms with potentials *f*(*x*) and *g*(*x*) located in quasiperiodic order defined by ratio of periods equal to *τ*. Thus, the resulting potential is


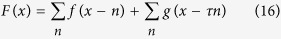


This potential as it was said can be represented as a slice of periodic function


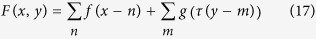


Using this image of potential, we will be able to use previously introduced method.

## Conclusions

This work proposed a new method of description quasiperiodic wave-functions of non-interacting electrons.

Equation which might be used for obtaining quasicrystals spectra in periodic boundaries is derived. Our method gives an ability easily get all the information about non-interacting electrons in arbitrary quasiperiodic potential.

It is essential that with derived equation we may describe both quasiperiodic and periodic materials^13^. Interfaces between are also might be studied. Since the generalization of the method and way of application is shown, results for real quasicrystalline materials can be obtained.

Nevertheless, I should emphasize that while the equation may clear up the picture with non-interacting particles, it is at least very difficult to apply it for interacting ones. Because the distance in physical space (on the slice) and in the “unit cell” is sufficiently different. However, very interesting case of weak-interacting particles with pair potential in form of *V*(*x*_1_, *x*_2_) = *Vδ*(*x*_1_ − *x*_2_) is one of the cases where one can use the method developed here.

## Additional Information

**How to cite this article**: Blinov, I. V. Periodic almost-Schrödinger equation for quasicrystals. *Sci. Rep.* **5**, 11492; doi: 10.1038/srep11492 (2015).
